# Construction of a polycystic ovarian syndrome (PCOS) pathway based on the interactions of PCOS-related proteins retrieved from bibliomic data

**DOI:** 10.1186/1742-4682-6-18

**Published:** 2009-09-01

**Authors:** Zeti-Azura Mohamed-Hussein, Sarahani Harun

**Affiliations:** 1 School of Biosciences and Biotechnology, Faculty of Science and Technology, Universiti Kebangsaan Malaysia, 43600, UKM Bangi, Selangor, Malaysia; 2 Centre for Bioinformatics Research, Institute of Systems Biology (INBIOSIS), Universiti Kebangsaan Malaysia, 43600, UKM Bangi, Selangor, Malaysia

## Abstract

Polycystic ovary syndrome (PCOS) is a complex but frequently occurring endocrine abnormality. PCOS has become one of the leading causes of oligo-ovulatory infertility among premenopausal women. The definition of PCOS remains unclear because of the heterogeneity of this abnormality, but it is associated with insulin resistance, hyperandrogenism, obesity and dyslipidaemia. The main purpose of this study was to identify possible candidate genes involved in PCOS. Several genomic approaches, including linkage analysis and microarray analysis, have been used to look for candidate PCOS genes. To obtain a clearer view of the mechanism of PCOS, we have compiled data from microarray analyses. An extensive literature search identified seven published microarray analyses that utilized PCOS samples. These were published between the year of 2003 and 2007 and included analyses of ovary tissues as well as whole ovaries and theca cells. Although somewhat different methods were used, all the studies employed cDNA microarrays to compare the gene expression patterns of PCOS patients with those of healthy controls. These analyses identified more than a thousand genes whose expression was altered in PCOS patients. Most of the genes were found to be involved in gene and protein expression, cell signaling and metabolism. We have classified all of the 1081 identified genes as coding for either known or unknown proteins. Cytoscape 2.6.1 was used to build a network of protein and then to analyze it. This protein network consists of 504 protein nodes and 1408 interactions among those proteins. One hypothetical protein in the PCOS network was postulated to be involved in the cell cycle. BiNGO was used to identify the three main ontologies in the protein network: molecular functions, biological processes and cellular components. This gene ontology analysis identified a number of ontologies and genes likely to be involved in the complex mechanism of PCOS. These include the insulin receptor signaling pathway, steroid biosynthesis, and the regulation of gonadotropin secretion among others.

## Background

Stein and Leventhal pioneered the study of Polycystic Ovary Syndrome (PCOS) in 1935 when they identified the abnormality in a small group of women with amenorrhea, hirsutism, obesity and histological evidence of polycystic ovaries [1]. Today, PCOS is a common endocrine disorder affecting 6.5-8.0% of all women of reproductive age [2]. There is no universal definition for this heterogeneous endocrine disorder [2]. However, during the 2003 Rotterdam Consensus workshop, PCOS was defined as a multi-system network of abnormalities that includes obesity, insulin resistance, hyperandrogenism, elevated luteinizing hormone (LH) concentrations, increased risk of type 2 diabetes mellitus, cardiovascular events and menstrual irregularities [3]. Insulin resistance is found in up to 70% of women with PCOS and 80% of the PCOS patients are hyperandrogenemic [4]. Several pathways are thought to be involved in PCOS, and these include steroid hormone synthesis [5,6], the insulin-signaling pathway [7] and gonadotrophin hormone action [8]. Mutation analyses, linkage studies and case-control association studies have been used to assess the roles of candidate genes from these pathways in PCOS [9]. CYP11A is a steroid synthesis gene that was found to be associated with PCOS and serum testosterone levels by a genetic polymorphism study [5]. A linkage analysis using PCOS patients revealed the involvement of a 5' region of the insulin gene that contains a variable number of tandem repeats (VNTRs) [10]. However, none of those genes are likely to be the key players in the pathogenesis of PCOS because its complexity and heterogeneity suggest the involvement of many genes as well as environmental factors [4,9].

Another genomic technique that has been widely used to investigate the mechanism of PCOS and to identify candidate PCOS genes is the microarray-based comparison of ovarian tissues (theca cells, follicular granulose cells, total ovarian tissue, and ovarian connective tissue) from PCOS patients with ovarian tissues from healthy controls [4]. The first PCOS microarray study was published by Wood and colleagues in 2003 [11]. They used theca cells from PCOS women and healthy controls as their samples and identified 244 differentially expressed genes. Their findings on the upregulation of GATA-6, which is involved in the transcription of CYP11A supported earlier linkage analyses [5]. Several other microarray analyses have helped shed light on the pathophysiology of PCOS. These results contributed to the dataset used in this study. The goal of this study was to obtain a clearer view of the mechanism of PCOS, since the definition of the abnormality remains unclear. Therefore we collated information on proteins related to PCOS, constructed a hypothetical network of interactions among PCOS-related proteins, and then inferred the function of a hypothetical protein that may be involved in PCOS.

## Methods

A number of previous studies, including mutation analyses, linkage studies and case-control association studies have identified 58 candidate PCOS genes [9]. In order to identify more proteins that may be related to PCOS, results from microarray analyses were used as a dataset in this study. These results were gathered from a literature search of various literature databases such as ScienceDirect  and PubMed  among others. Candidate proteins were then classified manually as either known proteins or hypothetical proteins. The sequences of the hypothetical proteins were analyzed in detail to shed light on their functions. BLAST  was used to run similarity searches on the hypothetical proteins to infer functional and evolutionary relationships between protein sequences. To gain further functional information, InterProScan  was used to search the protein sequences for motifs characteristic of previously described domains and protein families. Moreover, PROSCAN  was used to scan the protein sequences for sites and/or signatures contained in the PROSITE database. This tool is used to identify biologically relevant sites, patterns and profiles in a protein sequences.

All of the proteins identified by these methods were combined with the 58 PCOS-related proteins identified from the literature review. These proteins were then loaded into Cytoscape 2.6.1 [12] using the BioNetBuilder plugin. BioNetBuilder 2.0 [13] is an open-source network visualization platform. BioNetBuilder uses a variety of databases that include DIP (Database of Interacting Proteins), BIND (Biomolecular Interaction Network Database), HPRD (Human Protein Reference Database), KEGG (Kyoto Encyclopedia of Genes and Genomes) and MINT (Molecular Interaction Database) among others. However, since our study involves only proteins found in humans, only four databases were used: KEGG, HPRD, BIND and MINT. All of the collated proteins have their own UniProt ID and these were used as input for BioNetBuilder 2.0. Pathway construction with BioNetBuilder 2.0 usually takes several minutes depending on the amount of input loaded as well as the internet server used. BiNGO [14] was used to analyze the gene ontology in the PCOS network.

## Results and Discussion

Seven microarray analyses were identified in the scientific literature published between 2003 and 2007. The first paper was published by Wood and colleagues, who studied theca cells isolated from average-sized follicles of the ovaries of PCOS patients and normal women [11]. This same group conducted a similar study in 2004 but used theca cells treated with valproic acid (VPA) in order to assess the involvement of VPA with PCOS [15]. In the same year, two different PCOS microarray studies were published [16,17]. In 2005, scientists from Finland used cDNA microarrays to identify differentially expressed genes in ovarian connective tissue. [18]. The most recent study, published in 2007 by two different research groups, used two types of samples; omental adipose tissue [19] and oocyte samples [20] taken from PCOS patients. All of the differentially expressed genes identified by these microarray analyses are listed in Table 1. The differentially expressed genes were then identified and thoroughly analyzed. Any overlapping genes involved in more than one microarray study were unified. Moreover, the identified genes were compared with protein databases such as UniProt to gather their biological information such as their origin, function, domain and protein family, the ontology involved, their interactions and pathways as well as other information on their 3D structure that have been experimentally determined [21]. Thus, the overall number of genes was reduced and the remainders were classified as either known proteins or hypothetical proteins. The total number of proteins identified was 1081, and these consisted of 1066 known proteins and 15 hypothetical proteins. These proteins comprised the dataset used in the remainder of the study.

**Table 1:** Microarray analyses of PCOS samples from 2003 to 2007

**Microarray study**	**Number of differentially expressed genes**
The molecular phenotype of PCOS theca cells and new candidate genes defined by microarray analysis [11]	**244**
Valproate-induced alteration in human theca cell gene expression [15]	**199**
Abnormal gene expression profiles in human ovaries from polycystic ovary syndrome [16]	**135**
The molecular characteristics of PCOS defined by human ovary cDNA microarray [17]	**119**
Molecular profiling of polycystic ovaries for markers of cell invasion and matrix turnover [18]	**44**
Differential gene expression profile in omental adipose tissue in women with PCOS [19]	**63**
Molecular abnormalities in oocytes from women with PCOS revealed by microarray analysis [20]	**374**

**TOTAL**	**1178**

Sequence analyses were conducted to infer the function of each hypothetical protein. These analyses yielded numerous results. However, BLASTP analysis failed to identify any important functional or evolutionary relationship between the hypothetical proteins and known proteins. Moreover, most of the hypothetical proteins did not have any recognizable domains or protein family signatures in their sequences. Only one hypothetical protein (KIAA0247) had domain, family and superfamily associations in its protein sequence. The domain recognized is a sushi domain, also known as a complement control protein (CCP) module or short consensus repeat (SCR). Most of the hypothetical proteins contain casein kinase II phosphorylation sites and protein kinase C phosphorylation sites. Casein kinase II (CK-2) is a serine/threonine protein kinase whose activity is independent of cyclic nucleotides and calcium. CK-2 phosphorylates many different proteins. This pattern is found in most of its known physiological substrates [22]. Protein kinase C preferentially phosphorylates serine and threonine residues that are near C-terminal basic residues. The presence of additional basic residues at the N- or C-terminus of the target amino acid enhances the Vmax and Km of the phosphorylation reaction [23].

**Figure 1:**
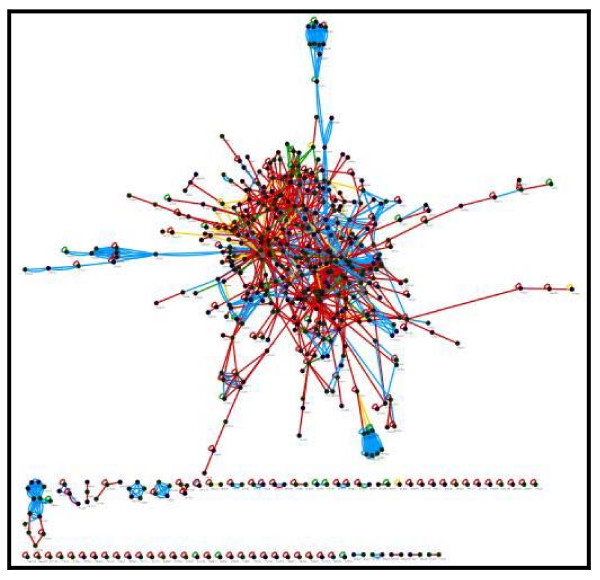
**The hypothetical PCOS pathway assembled by BioNetBuilder 2.0 in Cytoscape 2.6.1**. From the 1081 input genes, only 504 protein nodes and 1408 interactions among those proteins were assembled. Protein-protein interactions identified by the HPRD database (712) are represented in red. Interactions identified by the KEGG database (561) are represented in blue. Interactions from the MINT database (68) are represented in yellow. Interactions from the BIND database (67) are represented in green.

**Figure 2:**
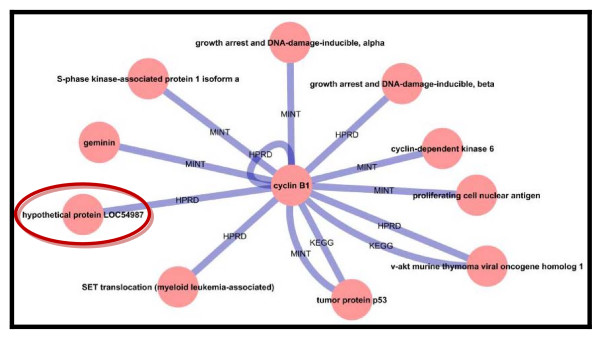
**Protein-protein interactions of the hypothetical protein**. The HPRD database identified an interaction between the hypothetical protein and cyclin B1. Cyclin B1 interacts with 9 other proteins including geminin, the tumor suppressor protein p53, and cyclin-dependent kinase 6 among others.

Several differentially expressed genes that were identified in more than one microarray analysis were chosen to be analyzed in detail in the protein network. The alpha actin 2 protein was found to be downregulated both by the Wood group in 2003 and by the Cortón group in 2007. Actins are usually involved in cell motility and are ubiquitously expressed in all eukaryotic cells. The HPRD database linked ACTA2 with SHBG, which is a protein frequently identified in linkage analyses of PCOS. SHBG expression tends to be reduced in PCOS patients due to their elevated insulin levels. Thus, decreased levels of alpha actin will lead to a reduced level of SHBG, which in turn increases the bioavailability of androgens [24], a feature of PCOS. A PCOS network was constructed with the BioNetBuilder 2.0 plugin in Cytoscape 2.6.1. The UniProt accession numbers of each protein from the dataset were used as input for the construction of the PCOS network. From the list of 1081 genes loaded into Cytoscape, 504 protein nodes and 1408 protein interactions were assembled and visualized. The interactions among those proteins were determined with the aid of four protein-protein interaction databases, including HPRD (Human Protein Reference Database), KEGG (Kyoto Encyclopedia of Genes and Genomes), BIND (Biomolecular Interaction Network Database) and MINT (Molecular Interaction Database). Figure 1 shows the resulting PCOS protein network. This network predicted that one of the PCOS hypothetical proteins, which is LOC54987 interacts with a cyclin (Figure 2). Like aurora kinase and actin binding protein, cyclin B1 is an APC (anaphase promoting complex) substrate [25]. APC is a key cell cycle regulator that both initiates anaphase and regulates mitotic exit [26]. Further analysis conducted on this hypothetical protein (LOC54987) shown the existence of a signal peptide region that cleaved at amino acid position of 19. A signal peptide on a protein indicates that this protein is destined either to be secreted or to be a membrane component. LOC54987 is a single-domain protein; identified as DUF domain (DUF866, PF05907). It is categorized into a group of hypothetical eukaryotic proteins of unknown function; where one member in this group has been determined its 3D structure (1ZSO, *Plasmodium falciparum *MAL13P1.257) [27] and share 25.9% identity with LOC54987. LOC 54987 is a conserved hypothetical protein with two CXXC motifs strongly conserved in all other family members. LOC54987 is also known as chromosome 1 ORF123, and is found differentially expressed in PCOS oocytes [20], but unfortunately there is no evidence that this sequence has been isolated. Based on our predicted PCOS protein-protein interaction network, LOC54987 forms direct interaction with cyclin B1 where in the cell cycle, B type cyclins are usually present during the G2 exit and mitosis phase. Cyclin B1 also associates with CDK1 [28], forms complexes that regulate a number of processes during the G2 exit [29], and also involves in the progression through mitosis [30]. Cyclin B1 is a major regulator in mammalian mitosis whereby the inhibition of cyclin B1 transcription by the p53 tumor suppressor may inhibit the G2/M transition in human cells [31], which supported the interaction of cyclin B1-p53 in this study. Another interaction partners which directly involved in cell cycle regulation are Gadd45 (growth arrest and DNA-damage-inducible) alpha and beta, geminin, proliferating cell nuclear antigen, CDK6, and S-phase kinase associated protein 1 isoform a; whilst SET translocation (myeloid leukemia-associated) is a multitasking protein which involved in apoptosis, transcription, nucleosome assembly and histone binding; and v-akt murine thymona viral oncogene homolog 1 is type of AKT that is capable of phosphorylating several known proteins. Based on this predicted interaction, LOC54987 can be identified as a new protein that functions as a cell-cycle regulator, due to its direct interaction with cyclin B1. As other proteins are widely studied, thus it is very interesting to further analyze LOC54987 experimentally to have detailed understanding on the protein itself and also to validate this predicted interaction.

**Figure 3:**
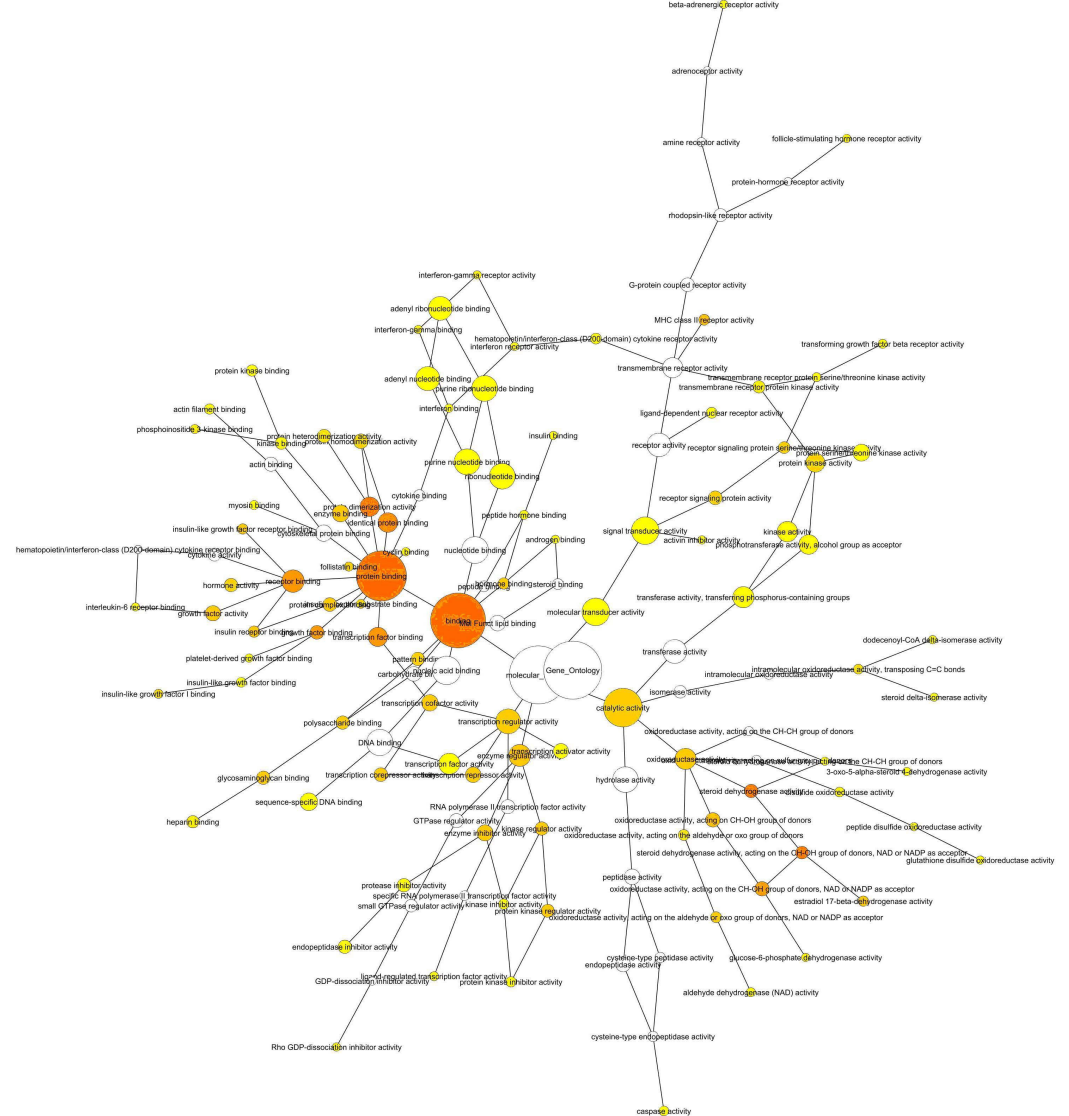
**Molecular function map**. Map of molecular functions associated with PCOS. Darker nodes refer to the significant ontologies of the dataset. The size is proportional to the number of genes that participate in that molecular function.

**Figure 4:**
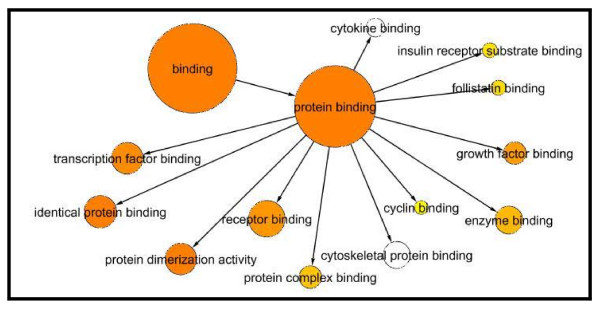
**Protein binding node**. The protein binding node is connected to 12 other nodes. The protein binding node is the most significant node because it encompasses 331 genes. Of the 468 genes in the PCOS molecular function network, 339 (72.4%) are involved in protein binding and are thereby linked to other protein nodes such as the transcription factors (34 genes), identical proteins (37 genes), protein dimerization (35 genes), receptors (50 genes), protein complexes (13 genes), cyclins (2 genes), enzymes (24 genes), growth factors (13 genes), follistatins (3 genes), and insulin receptor substrates (3 genes).

The PCOS network was then analyzed with the Biological Networks Gene Ontology (BiNGO) program to identify ontologies involved in the protein network. BiNGO is one of the plugins for Cytoscape 2.6.1. Three ontologies (cellular component, molecular function and biological process) were identified in the BiNGO analysis. Figure 3 shows the results from the BiNGO analysis regarding molecular function. The binding node is the most significant node as it involves 415 of the 468 proteins. The binding node encompasses multiple types of molecular interactions, including protein binding, nucleic acid binding, peptide binding, pattern binding, carbohydrate binding and nucleotide binding. Detailed results are displayed in Figure 4. Other molecular function nodes that may play an important role in the pathogenesis of PCOS include steroid dehydrogenase activity (12 genes) and estradiol 17-beta-dehydrogenase activity (5 genes), among others. The molecular function mode of BiNGO identified three genes (INHBA, ACVR1 and ACVR2A) involved in follistatin binding. Follistatin was also implicated in PCOS by linkage analysis [9]. BiNGO identified several biological processes that maybe involved with PCOS including lipid metabolism (40 genes), regulation of apoptosis (56 genes), insulin receptor signaling (9 genes), steroid biosynthesis (13 genes) and the regulation of gonadotropin secretion (3 genes). Of the 473 genes in biological process genes identified by BiNGO, 40 were involved with lipid metabolism. One of these genes, ALOX15, is a lipoxygenase that was found to be upregulated in omental fat of PCOS patients [19]. ALOX15 may be involved with insulin resistance since a number of lypoxygenase-oxidized fatty acids become leukotrienes, which contribute to the chronic inflammatory condition of PCOS [19]. Laboratory findings support this idea as the inhibition of lipoxygenases was found to enhance the action of insulin in rat models of insulin resistance and type 2 diabetes [32].

## Conclusion

Information from genomic analysis is ideally suited to elucidating the mechanism of complex syndrome such as PCOS. Therefore, we constructed a dataset composed of information from microarray analyses and other genomic studies of PCOS patients. This dataset, which consists of 1081 candidate genes, was used to construct a PCOS network. This network contains 504 protein nodes and 1408 interactions between those proteins. The network predicted that a hypothetical protein whose function was previously unknown interacts with cyclin B1. Thus this hypothetical protein may be involved in the cell cycle. The network also identified a number of molecular functions and biological processes likely to be involved in PCOS. These include steroid dehydrogenase activity, estradiol 17-beta-dehydrogenase activity, lipid metabolism, regulation of apoptosis, the insulin receptor signaling pathway, steroid biosynthesis and the regulation of gonadotropin secretion. The genes involved in these molecular functions and biological processes were then categorized as genes likely to have important roles in the mechanism of PCOS.

## Competing interests

The authors declare that they have no competing interests.

## Authors' contributions

SH performed the research and drafted the manuscript. ZAMH formulated the study, gave informative suggestions upon the research and refined the manuscript. All authors read and approved the final manuscript.

## References

[B1] Stein IL, Leventhal ML (1935). Amenorrhea associated with bilateral polycystic ovaries. Am J Obstet Gynecol.

[B2] Goodarzi MO, Azziz R (2006). Diagnosis, epidemiology, and genetics of the polycystic ovary syndrome. Best Prac Res Clin Endocrinol Metab.

[B3] Rotterdam ESHRE/ASRM--Sponsored PCOS Concensus Workshop Group (2004). Revised 2003 consensus on diagnostic criteria and long-term health risks related to polycystic ovary syndrome. Fertil Steril.

[B4] Goodarzi MO (2008). Looking for polycystic ovary syndrome genes: rational and best strategy. Sem Rep Med.

[B5] Gharani N, Waterworth DM, Batty S, White D, Gilling-Smith, Conway GS, McCarthy M, Franks S, Williamson R (1997). Association of the steroid synthesis gene CYP11a with polycystic ovary syndrome and hyperandrogenism. Hum MolGenet.

[B6] Carey AH, Waterworth D, Patel K, White D, Little J, Novelli P, Franks S, Williamson R (1994). Polycystic ovaries and premature male pattern baldness are associated with one allele of the steroid metabolism gene CYP17. Hum Mol Genet.

[B7] Dunaif A, Segal KR, Shelley DR, Green G, Dobrjansky A, Licholai T (1992). Evidence of distinctive and intrinsic defects in insulin action in polycystic ovary syndrome. Diabetes.

[B8] Frank S (1995). Polycystic ovary syndrome. N Eng J Med.

[B9] Urbanek M, Legro RS, Driscoll DA (1999). Thirty-seven candidate genes for polycystic ovary syndrome: strongest evidence for linkage is with follistatin. Proc Nat Acad USA.

[B10] Waterworth DM, Bennett ST, Gharani N, McCarthy MI, Hague S, Batty S, Conway GS, White D, Todd JA, Franks S (1997). Linkage and association of insulin gene VNTR regulatory polymorphism with polycystic ovary syndrome. Lancet.

[B11] Wood JR, Nelson-Degrave VL, Ho C, Jansen E, Wang CY, Urbanek M, McAllisters JM, Mosselman S, Strauss JF (2003). The molecular phenotype of polycystic ovary syndrome (PCOS) theca cells and new candidate PCOS genes defined by microarray analysis. J Biol Chem.

[B12] Shannon P, Markiel A, Ozier O, Baliga NS, Wang JT, Ramage D, Amin N, Schwikowski B, Ideker T (2003). Cytoscape: a software environment for intergrated models of biomocular interaction networks. Genome Res.

[B13] Avila-Campilo I, Drew K, Lin J, Reiss DJ, Bonneau R (2007). BioNetBuilder: automatic integration of biological networks. Bioinformatics.

[B14] Maere S, Heymans K, Kuiper M (2005). BiNGO: a Cytoscape plugin to access overrepresentation of Gene Onology categories in Biological Networks. Bioinformatics.

[B15] Wood JR, Nelson-Degrave VL, Jansen E, McAllisters JM, Mosselman S, Strauss JF (2004). Valproate-induced alterations in human theca cell gene expression: clues to the association between valproate use and metabolic side effects. Physiol Genomics.

[B16] Jansen E, Laven JS, Dommerholt HB, Polman J, van Rijt C, Hurk C van den, Westland J, Mosselman S, Fauser BC (2004). Abnormal gene expression profiles in human ovaries from polycystic ovary syndrome patients. Mol Endocrinol.

[B17] Diao FY, Xu M, Hu Y, Li J, Xu Z, Lin M, Wang L, Zhou Y, Zhou Z, Liu J, Sha J (2004). The molecular characteristics of polycystic ovary syndrome (PCOS) ovary defined by human ovary cDNA microarray. J Mol En Endocrinol.

[B18] Oksjoki S, Soderstrom M, Inki P, Vuorio E, Anttila L (2005). Molecular profiling of polycystic ovaries for markers of cell invasion and matrix turnover. Fertil Steril.

[B19] Cortón M, Botella-Carretero JI, Benguria A, Villuendas G, Zaballos A, San Milan JL, Escobar-Morreale HF, Peral B (2007). Differential gene expression profile in omental adipose tissue in women with polycystic ovary syndrome. J Clin Endocrinol Metab.

[B20] Wood JR, Dumesic DA, Abbott DH, Strauss JF (2007). Molecular abnormalities in oocytes from women with polycystic ovary syndrome revealed by microarray analysis. J Clin Endocrinol Metab.

[B21] Jain E, Bairoch A, Duvaud S, Phan I, Redaschi N, Suzek BE, Martin MJ, McGarvey P, Gasteiger E (2009). Infrastructure for the life sciences: design and implementation of the UniProt website. BMC Bioinformatics.

[B22] Pinna LA (1990). Casein kinase 2: an 'eminence grise' in cellular regulation?. Biochim Biophys Acta.

[B23] Kishimoto A, Nishiyama K, Nakanishi H, Uratsuji Y, Nomura H, Takeyama Y, Nishizuka Y (1985). Studies on the phosphorylation of myelin basic protein by protein kinase C and adenosine 3':5'-monophosphate-dependent kinase. J Biol Chem.

[B24] Amato P, Simpson JL (2004). The genetics of polycystic ovary syndrome. Best Prac Res Cl Ob.

[B25] Dephoure N, Zhou C, Villen J, Beausoleil SA, Bakalarski CE, Elledge SJ, Gygi SP (2008). A quantitative atlas of mitotic phosphorylation. Proc Nat Acad USA.

[B26] Kraft C, Herzog F, Gieffers C, Mechtler K, Hagting A, Pines J, Peters J (2003). Mitotic regulation of human anaphase-promoting complex by phosphorylation. Eur Mol Biol Org.

[B27] Holmes MA, Buckner FS, Van Voorhis WC, Mehlin C, Boni E, Earnest TN, DeTitta G, Luft J, Lauricella A, Anderson L, Kalyuzhniy O, Zucker F, Schoenfeld LW, Hol WGJ, Merritt EA (2006). Structure of the conserved hypothetical protein MAL13P1.257 from Plasmodium falciparum. Acta Cryst.

[B28] Coqueret O (2003). New targets for viral cyclins. Cell Cycle 2.

[B29] Nigg EA (2001). Mitotic kinases as regulators of cell division and its checkpoints. Nat Rev Mol Cell Biol.

[B30] Malumbres M, Barbacid M (2005). Mammalian cyclin-dependent kinases. Trend Biochem Sci.

[B31] Innocente SA, Abrahamson JL, Cogswell JP, Lee JM (1999). p53 regulates a G2 checkpoint through cyclin B1. Proc Natl Acad Sci USA.

[B32] Reed MJ, Meszaros K, Entes LJ, Claypool MD, Pinkett JG, Brignetti D, Luo J, Khandwala A, Reaven GM (1999). Effect of masoprocol on carbohydrate and lipid metabolism in a rat model of Type II diabetes. Diabetologia.

